# Disparity in Risk Factor Severity for Early Childhood Blood Lead among Predominantly African-American Black Children: The 1999 to 2010 US NHANES

**DOI:** 10.3390/ijerph17051552

**Published:** 2020-02-28

**Authors:** Deniz Yeter, Ellen C. Banks, Michael Aschner

**Affiliations:** 1 Independent Researcher, Kansas City, KS 66104, USA; 2 Daemen College, Amherst, NY 14226, USA; ebanks@daemen.edu; 3 Department of Molecular Pharmacology, Albert Einstein College of Medicine, Bronx, NY 10461, USA; michael.aschner@einsteinmed.org

**Keywords:** African-American ethnicity, environmental justice, epidemiology, lead (Pb) exposure, metal toxicology, racial disparity, pediatrics, public health, safe housing, socio-economics

## Abstract

There is no safe detectable level of lead (Pb) in the blood of young children. In the United States, predominantly African-American Black children are exposed to more Pb and present with the highest mean blood lead levels (BLLs). However, racial disparity has not been fully examined within risk factors for early childhood Pb exposure. Therefore, we conducted secondary analysis of blood Pb determinations for 2841 US children at ages 1–5 years with citizenship examined by the cross-sectional 1999 to 2010 National Health and Nutrition Examination Survey (NHANES). The primary measures were racial disparities for continuous BLLs or an elevated BLL (EBLL) ≥5 µg/dL in selected risk factors between non-Hispanic Black children (*n* = 608) and both non-Hispanic White (*n* = 1208) or Hispanic (*n* = 1025) children. Selected risk factors included indoor household smoking, low income or poverty, older housing built before 1978 or 1950, low primary guardian education <12th grade/general education diploma (GED), or younger age between 1 and 3 years. Data were analyzed using a regression model corrected for risk factors and other confounding variables. Overall, Black children had an adjusted +0.83 µg/dL blood Pb (95% CI 0.65 to 1.00, *p* < 0.001) and a 2.8 times higher odds of having an EBLL ≥5 µg/dL (95% CI 1.9 to 3.9, *p* < 0.001). When stratified by risk factor group, Black children had an adjusted 0.73 to 1.41 µg/dL more blood Pb (*p* < 0.001 respectively) and a 1.8 to 5.6 times higher odds of having an EBLL ≥5 µg/dL (*p* ≤ 0.05 respectively) for every selected risk factor that was tested. For Black children nationwide, one in four residing in pre-1950 housing and one in six living in poverty presented with an EBLL ≥5 µg/dL. In conclusion, significant nationwide racial disparity in blood Pb outcomes persist for predominantly African-American Black children even after correcting for risk factors and other variables. This racial disparity further persists within housing, socio-economic, and age-related risk factors of blood Pb outcomes that are much more severe for Black children.

## 1. Introduction

There is no safe detectable level of lead (Pb) in the blood of young children, which is a position that has recently been officially adopted by the US Centers for Disease Control (CDC) [1] and the American Academy of Pediatrics (AAP) [2]. Exposure to Pb in young children largely impacts the cerebellum, hippocampus, and prefrontal cerebral cortex [3] while greater exposure during early childhood later results in decreased brain volume in adulthood [4]. Even at lower levels, neurodevelopmental effects of pre or postnatal Pb exposure include lasting neuropsychological deficits in attention, behavior, cognition, intelligence, and memory [5,6,7,8,9,10,11,12,13,14]. International pooled analysis of children 6–24 months of age observed a loss of 1.88 intelligence quotient (IQ) points for each doubling of blood lead levels (BLLs) beginning at 2 µg/dL [15,16,17]. Recent meta-analysis demonstrated that even slight increases in BLLs below 3 µg/dL are still significantly associated with a greater risk of presenting with symptoms of attention-deficit/hyperactivity disorder (ADHD) among children 5–12 years of age [18]. Infants and preschool-aged children are at higher risk of Pb exposure in part due to their increased body surface area, increased heart and respiratory rates, the ingestion and inhalation of contaminated dust or soil from greater hand-to-mouth activity, pica, crawling, and their low stature to the ground [19]. Exposure to Pb during early childhood that results in blood Pb concentrations between 30 and 100 µg/dL leads to the development of clinical lead poisoning, which is characterized by a severe hemolytic anemia, nephropathy, and encephalopathy proceeding to coma and death beginning at BLLs of approximately 100 to 150 µg/dL [20,21,22,23].

In the United States, the hazards of Pb exposure in children particularly during early childhood were first widely recognized with the phasing out of added Pb to gasoline since 1973 and restrictions upon maximum Pb concentrations present within paint since 1977. Bans on the new installation of leaded plumbing fixtures and piping used for drinking water began in 1986. These restrictions upon the predominant sources of early childhood Pb exposure led to the dramatic decrease in mean BLLs for US children <6 years of age, which was 16 µg/dL in 1976 to 1980 and has since fallen to 2 µg/dL in 2007 to 2010 [24,25,26,27,28]. Similar early childhood BLL reductions are reported throughout the world in both developed and developing nations—particularly after the removal of leaded gasoline [29]. In the present day, early childhood Pb exposure in the US mostly occurs from deteriorated leaded paint in older housing built before 1978 or particularly 1950, contaminated soil, air pollution, Pb-emitting industries, automobiles, traffic, contaminated drinking water predominantly from the deterioration of leaded household service lines, and passive exposure to tobacco smoke [30,31,32,33,34,35].

According to current CDC guidelines, a BLL ≥5 µg/dL is regarded to be an elevated BLL (EBLL) for young children [28]. The CDC developed a risk screening questionnaire in 1991 to identify EBLLs ≥10 µg/dL that should prompt public health interventions [36], although recent systematic reviews have found that both the 1991 CDC questionnaire and others only perform a little better than if left to pure chance alone [30,37]. Since 1998, federal Medicaid rules (§5123.2.D.1.) mandate that a child who is enrolled in Medicaid or the Children’s Health Insurance Program (CHIP) through title XIX or XXI funding must undergo blood Pb testing at 12 and also 24 months of age that cannot be supplemented by a risk screening questionnaire. If both of these blood tests are missed, the child must receive blood Pb testing at least once by 72 months of age. The AAP currently recommends targeted screening of children 12 to 24 months of age living in areas where ≥25% of housing is built before 1960 or where ≥5% of children present with an EBLL ≥5 μg/dL, children who reside in or visit homes or child care facilities with identified lead hazards, or children who live in a home built before 1960 that is in poor condition or was recently renovated [30]. The American College of Preventative Medicine (ACPM) further recommends screening of children at 1 year of age if they are enrolled in the federal Women, Infants, and Children (WIC) supplemental food program. Other risk factors that are commonly used in screening for early childhood blood Pb include older housing built before 1978 or 1950, identified household Pb hazards, indoor smoking, poor socio-economic status including lower family income or primary guardian educational attainment, younger age, anemia, and race or ethnicity [30,31,32,33,34].

Significant nationwide racial disparity in blood Pb has been documented among predominantly African-American Black children since 1976 [24,25,26,27,28], while ethnic disparity for Hispanic children is no longer seen at the national level compared to White children since the 1990s. In the present day, Black children continue to have the highest average BLLs in the US compared to either non-Hispanic White or Hispanic children [38]. Black race remains a strong independent predictor of more blood Pb throughout childhood [39,40,41,42,43,44,45,46]. Geospatial analyses have demonstrated that increasing concentrations of exposure to Pb from the air, soil, water, or industrial releases are associated with early childhood BLLs [47,48,49,50,51,52,53,54], while an increasing percent Black population is a strong predictor for higher blood Pb among young children in US cities, states, and nationwide across the US [32,33,34,39,40,53,55,56,57,58,59,60,61,62,63]. Black children in the US are exposed to more Pb from their environment [47,48,49,50,64,65,66,67,68,69]. Household Pb dust contamination, poorer conditions of interior paint, and poor or inadequate housing are far worse in outcome for Black children [67,68]. Ongoing leaded paint hazards in older US homes still requiring cost-prohibitive remediation disproportionately affect children who are Black [68,69]. Black children throughout the US are exposed to significantly more Pb from the ambient air [47,48]. In the Detroit metropolitan area, Pb-emitting industries are both primarily located within and currently relocating to predominantly Black neighborhoods irrespective of income levels [48]. Greater intensity of Pb soil contamination is associated with increasing percent Black populations in Alabama, Louisiana, and South Carolina respectively where Pb-contaminated soils are largely confined to neighborhoods that are predominantly Black [64,65,66].

Increasing racial segregation and lower socio-economic status are associated with higher blood Pb among young Black children in the Detroit metropolitan area [40]. Black children present with the highest BLLs during early childhood throughout Marion County, Indiana, where remarkably higher rates of an EBLL ≥10 µg/dL are mostly secluded to the predominantly Black urban core of the City of Indianapolis [39]. For the City of St. Louis in Missouri, the overall blood Pb burden among young children is disproportionately accounted for by poor Black children residing in predominantly Black neighborhoods [70]. Recent water crises that involved Washington D.C. beginning in 2001 and Flint, Michigan, beginning in 2014 had each resulted in epidemic increases in early childhood blood Pb that were both secluded to these predominantly Black cities while the surrounding predominantly White areas were spared [49,50]. Furthermore, young Black children in the US residing below the poverty level are twice as likely to present with an EBLL ≥10 µg/dL than their poor White counterparts at the national level [71]. However, Black racial disparities among US children have not been examined for other risk factors of Pb exposure during early childhood. Therefore, we examined racial disparities for early childhood blood Pb outcomes within risk factor subgroups related to housing conditions, socio-economics, and younger age that were selected after a review of the literature.

## 2. Materials and Methods

### 2.1. Study Sample

The National Health and Nutrition Examination Survey (NHANES) is a cross-sectional study conducted by the CDC at the direction of the Department of Health and Human Services (HHS) to assess the current health and nutritional status of individuals residing inside the US. The NHANES collects a nationally representative sample of the non-institutionalized population that is then later de-identified and made freely available online (www.cdc.gov/nchs/nhanes.htm) [72]. Additional limited datasets and stored biological specimens can be accessed from the NHANES at their Research Data Centers or biorepositories following approval for access. Written informed consent is obtained by the NHANES from each participant or the primary guardian when involving a child participant, and its study protocols were approved by the National Center for Health Statistics (NCHS) research ethics review board. For this study, publicly-available data were retrieved from the NHANES for our own secondary analyses that do not require an additional institutional review board (IRB) approval.

**Figure 1:**
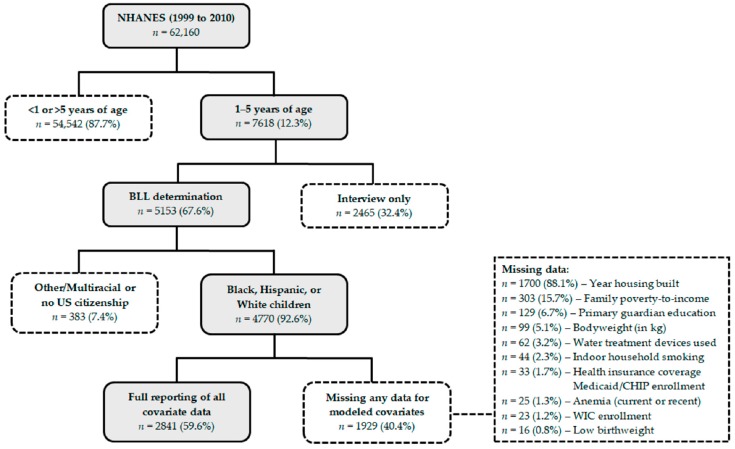
Case selection schematic for exclusion or inclusion in the studied sample.

As shown in Figure 1, data were available for 4770 US children aged 1–5 years examined by the 1999 to 2010 NHANES after excluding 383 children (7.4%) identifying as Other/Multiracial (*n* = 285) or without US citizenship (*n* = 101). Covariate data of the retrieved cases were also obtained for age in years, binary gender, race/ethnicity, family poverty-to-income ratios, primary guardian educational attainment, indoor household smoking, the year housing was built, 2 year survey cycles, bodyweight, birthweight, low birthweight, blood hemoglobin level, anemia treatment within the last 3 months, health insurance coverage, and use of household water treatment devices. After excluding 1929 cases for any missing covariate data (40.4%), a total of 2841 children were included in regression model analyses. Lastly, the number of cigarettes smoked inside of the home per day was included as an additional covariate in the stratified regression model analysis for children who are all exposed to indoor household smoking, which resulted in a further loss of 16 out of 449 cases (3.6%) from missing data in this model only.

### 2.2. Blood Lead

Blood Pb concentrations were determined in the whole blood using a multi-element atomic absorption spectrometer (AAS) between 1999 and 2002, while BLLs since 2003 have been determined in the whole blood using inductively coupled plasma–mass spectrometry (ICP–MS). The lower limit of detection (LLOD) for blood Pb concentrations was 0.3 µg/dL from 1999 to 2004 and 0.25 µg/dL from 2005 to 2010. Undetectable levels of Pb in the blood were supplemented by the NHANES with a value equal to the lower detection limit divided by the square root of two. Continuous BLLs were rounded to the first decimal place to maintain significant figures. EBLLs ≥5 µg/dL were assigned by using the current CDC guideline of rounding BLLs to one decimal place so that measurements ≥4.5 µg/dL are identified [21]. Children who are younger than 12 months of age are not included in blood sample draws by the NHANES.

### 2.3. Covariates

For each child participant, the NHANES asks the household to select a reference member of the household considered to be the primary guardian for the child. The reference household member then completes questionnaires on behalf of the younger children. Poverty-to-income ratios (PIR) are determined on a scale of 0.00 to an upper limit of 5.00, which represent the total household income divided by federal HHS-adjusted poverty level guidelines that are specific to the family size, state of residence, and reporting year. Family PIRs were segmented by poverty (<100% of the poverty level), low income (100 to 199% of the poverty level), or middle to upper class (≥200% of the poverty level). Bodyweights were measured by a trained NHANES examiner. Birthweights were documented in pounds (lbs.) and ounces (oz.) from the questionnaire, while low birthweight was defined as <5 lbs. 8 oz. at birth. If birthweight was missing, questionnaire data as to whether or not the child weighed less than 5.5 lbs. were instead used. Blood hemoglobin was determined from a complete blood count (CBC) assessed by a Beckman Coulter MAXM instrument. Age-appropriate childhood anemia cases were identified using the current World Health Organization (WHO) guidelines [73]. Furthermore, children were categorized as anemic if they had received diet modifications or other treatments for anemia within the last 3 months. Data for the remaining variables used in regression model analyses were determined through questionnaire responses.

### 2.4. Statistical Analysis

Statistical significance was defined as a *p*-value ≤ 0.05 in any performed testing. Simple means of continuous BLLs were examined using independent T-tests for two categorical variables, ANOVA for three or more categorical variables, or Pearson correlation tests for continuous variables. Post-hoc analysis in ANOVA was used to determine statistical significance for covariates by comparing them to the first category as a reference. Simple means of rates for an EBLL ≥5 µg/dL were examined in chi-square tests. Regression analysis of racial disparity in early childhood blood Pb was conducted using SPSS version 23 (SPSS, Inc., Chicago, IL, USA) before and after adjusting for covariates. Linear regression model was used to analyze continuous BLLs and logistic regression was used to analyze categorical rates of EBLLs ≥5 µg/dL. Further analyses of Black racial disparity were then stratified by selected risk factors for early childhood Pb exposure that included indoor household smoking, low income or poverty, lower primary guardian education, older housing, and younger age. Covariates included in adjusted multiple regression model analysis were other risk factors, survey years, binary gender, bodyweight, low birthweight, anemia, health insurance coverage, Medicaid/CHIP or WIC enrollment, and the use water treatment devices inside the home. Models stratified by risk factor included an additional related covariate of either age in months, primary guardian education less than 9th grade, poverty-to-income ratios, the number of cigarettes smoked inside the home per day (1 to 40 or more), or housing built before 1940 or 1960. Lastly, NHANES exam weights were used to plot data and provide values for adjusted outcomes from each regression model that is presented in the graphical representations.

## 3. Results

### 3.1. Descriptive Statistics and Missing Data

Simple means of continuous BLLs and rates of an EBLL ≥5 µg/dL for risk factors and covariates used in later regression models are shown in Table 1. The highest significant mean BLLs and rates of an EBLL ≥5 µg/dL were observed for older housing built before 1950 (3.03 µg/dL; 15.8%), indoor household smoking (2.83 µg/dL; 13.4%), non-Hispanic Black children (2.81 µg/dL; 12.0%), poverty (2.57 µg/dL; 10.5%), the 1999 to 2002 survey years (2.56 µg/dL; 10.9%), 1 year of age (2.49 µg/dL; 10.8%), and anemia for EBLLs ≥5 µg/dL only (12.8%). There were no significant associations in blood Pb between males and females, children 3 and 4–5 years of age, and White and Hispanic children.

As shown in both Figure 1 and Table 1, the variable with the most missing data was for the year housing was built (64.4% reporting data; 88.1% of missing data) and was far more pronounced than other covariates with missing data (93.6 to 99.7% reporting data; 0.8 to 15.7% of missing data). Cases that were excluded for this covariate predominantly involved responses in which the age of housing was unknown (*n* = 1646) as opposed to refused (*n* = 10) or missing (*n* = 44) responses from the primary guardians. Therefore, our modeled sample with full reporting of the covariate data (59.6% reporting) is largely dependent upon the recall of housing age by responding primary guardians for children participating in the NHANES. Previous analysis of missing data from the 1999 to 2010 NHANES survey period found that the recall of the year housing was built is significantly dependent on home ownership and is much lower among respondents for Black or Hispanic children [41]. As shown in Table S1, our greatest loss of cases subsequently resulted from missing data for Hispanic children and particularly Black children. This led to the inclusion of a much larger sample of White children for the study. Lastly, exclusion for missing data significantly decreased continuous BLLs and rates of an EBLL ≥5 µg/dL overall, which then disproportionately impacted Black children the most.

### 3.2. Full Multiple Regression Model

Adjusted coefficients and adjusted odds ratios (AORs) from the full multiple regression model corrected for covariates are shown in Table 2. Significantly higher continuous BLLs and rates of an EBLL ≥5 µg/dL were observed for each selected risk factor examined except for 3 years of age, while lower primary guardian education was only significantly associated with higher continuous BLLs but not rates of an EBLL ≥5 µg/dL. Blood Pb was significantly lower for both of the later survey years following 1999 to 2002. Children enrolled in either Medicaid/CHIP or WIC did not present with any significant differences in blood Pb. Children with health insurance coverage, increasing bodyweight, or who reside in households using water treatment devices presented with significantly lower BLLs only but not with rates of an EBLL ≥5 µg/dL. Current or recent anemia within the last 3 months was significantly associated only with a higher rate of an EBLL ≥5 µg/dL but not with continuous BLLs.

The highest significant increases in adjusted BLLs and rates of an EBLL ≥5 µg/dL were observed for older housing built before 1950 (+1.22 µg/dL; AOR = 7.1), non-Hispanic Black children compared to Hispanic children (+0.92 µg/dL; AOR = 3.5), children 1 year of age (+0.57 µg/dL; AOR = 3.4), and poverty (+0.56 µg/dL; AOR = 2.5). Smaller yet significant increases in continuous BLLs were seen for uninsured children (+0.26 µg/dL) or those with a primary guardian who did not attain a high school diploma or GED (+0.22 µg/dL). Smaller significant decreases in continuous BLLs were also observed for increasing bodyweight (−0.02 µg/dL per kg) and children in households that use water treatment devices (−0.21 µg/dL). Rates of an EBLL ≥5 µg/dL between White and Hispanic children did not significantly differ and were only marginally different (*p* = 0.070). When compared to White children alone, Black children had an adjusted 2.3 times higher odds of presenting with an EBLL ≥5 µg/dL (*p* < 0.001). As shown in Table 3, Black children presented with an adjusted 0.83 µg/dL more blood Pb and had an adjusted 2.8 times higher odds of presenting with an EBLL ≥5 µg/dL (*p* < 0.001) compared to both White and Hispanic children.

### 3.3. Risk Factors in Stratified Multiple Regression Models

As shown in Table 3, racial disparity for Black children compared to White or Hispanic children was further examined within each selected risk factor subgroup by stratifying the regression model analysis to an individual risk factor subgroup. Overall racial disparity from the full regression model was used for comparison to racial disparity in stratified regression models. Additional results were then weighted to estimate nationally representative averages of continuous BLLs or rates of an EBLL ≥5 µg/dL along with their weighted outcomes in multiple regression model analysis.

#### 3.3.1. Continuous Blood Lead Level

**Figure 2:**
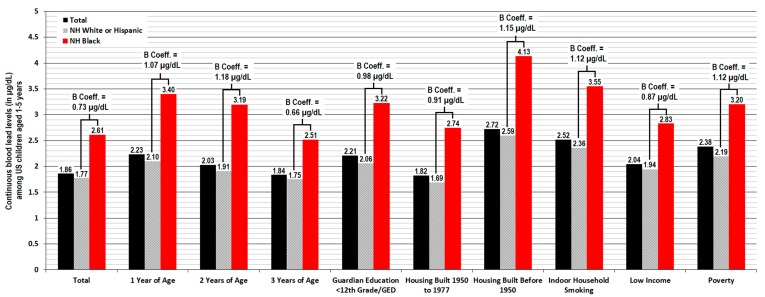
Weighted estimates of means and adjusted unstandardized coefficients for continuous blood lead levels (BLLs) within the general US population or risk factor groups among children 1–5 years of age with US citizenship—the 1999 to 2010 NHANES.

Significant racial disparity was observed for continuous BLLs in each risk factor subgroup both before and after controlling for covariates in the regression model (*p* < 0.001 respectively), which resulted in Black children having an adjusted 0.73 to 1.41 µg/dL more blood Pb. The most pronounced racial disparity in adjusted outcomes was seen at 2 years of age (+1.41 µg/dL, 95% CI 1.01 to 1.81, *p* < 0.001), for exposure to indoor household smoking (+1.30 µg/dL, 95% CI 0.79 to 1.80, *p* < 0.001), for children residing in poverty (+1.22 µg/dL, 95% CI 0.89 to 1.58, *p* < 0.001), and older housing built before 1950 (+1.22 µg/dL, 95% CI 0.54 to 1.90, *p* < 0.001). Racial disparity for continuous BLLs continued to remain significant but decreased in each modeled risk factor subgroup after controlling for other variables, which accounted for an average 13.1% (2.8 to 28.2%) reduction in the outcomes. Weighted results for nationally representative means of continuous BLLs are shown in Figure 2, in addition to weighted analyses from linear regression models.

#### 3.3.2. Elevated Blood Lead Level

**Figure 3:**
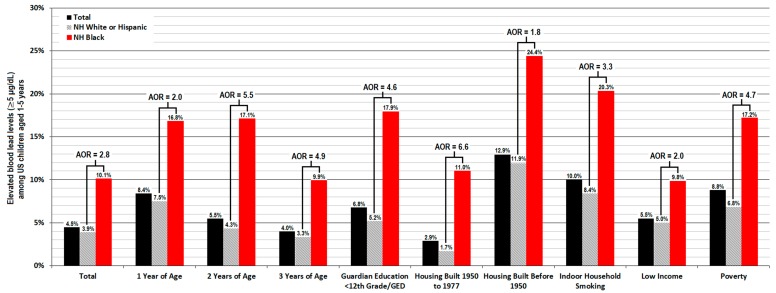
Weighted estimates of means and adjusted odds ratios (AORs) for rates of an elevated blood lead level (EBLL) ≥5 µg/dL within the general US population or risk factor groups among children 1–5 years of age with US citizenship—the 1999 to 2010 NHANES.

Significant racial disparity was observed for rates of an EBLL ≥5 µg/dL within each risk factor subgroup both before and after controlling for covariates in the regression model (*p* ≤ 0.05 respectively), which resulted in Black children having a 1.8 to 5.6 times higher odds of presenting with an EBLL ≥5 µg/dL. The most pronounced racial disparity in adjusted outcomes was seen for children residing in housing built between 1950 and 1977 (AOR = 5.6, 95% CI 2.8 to 11.2, *p* < 0.001), children 2 years of age (AOR = 5.3, 95% 2.6 to 10.7, *p* < 0.001), children 3 years of age (AOR = 4.7, 95% CI 1.7 to 13.1, *p* = 0.003), and children residing in poverty (AOR = 4.1, 95% CI 2.3 to 7.0, *p* < 0.001). Racial disparity for rates of an EBLL ≥5 µg/dL tended to increase after correcting for covariates in six out of nine models for individual risk factor subgroups. Weighted results for nationally representative averages of rates for an EBLL ≥5 µg/dL are shown in Figure 3, in addition to weighted analyses in logistic regression models.

## 4. Discussion

### 4.1. Persistent Racial Disparity in Risk Factors

In our current study, young predominantly African-American Black children had significantly worse outcomes for blood Pb than their White or Hispanic peers even after correcting for risk factors and other variables, which is in agreement with the published literature and also previous studies that examined this same 1999 to 2010 NHANES dataset [28,38,41]. We found that Black race was the second strongest predictor for worse blood Pb outcomes during early childhood following the risk of residing in pre-1950 housing—as evidenced by their β and Wald coefficients—while Black children experienced the second highest means for both continuous BLLs and EBLL rates ≥5 µg/dL during early childhood. Our further analyses using regression models that were stratified by individual risk factors for early childhood Pb exposure demonstrated that this racial disparity in blood Pb outcomes persisted for Black children within each examined risk factor to varying degrees. To our knowledge, this study is the first to examine racial disparities within risk factors for early childhood Pb exposure after adjusting for other risk factors and covariates. The most pronounced disparities were observed for Black children 2 to 3 years of age, those living in poverty or older housing built from 1950 to 1977, and those with a primary guardian who had not received a high school diploma or GED. Previous examination of the nationally representative 1988 and 1994 NHANES dataset found that Black children below the poverty level were more than twice as likely to present with an EBLL ≥10 µg/dL than their poor White peers from the simple mean differences [71]. In our current study of the 1999 to 2010 NHANES data, we observed that Black children living in poverty had a 4-fold higher odds of presenting with an EBLL ≥5 µg/dL compared to their White or Hispanic peers at the national level even when we rigorously controlled for other risk factors and confounding variables.

Therefore, these racial disparities in early childhood blood Pb outcomes do not appear to result from Black children simply having a higher rate of risk factors for early childhood Pb exposure. It instead seems that the severity of blood Pb outcomes related to risk factors for early childhood Pb exposure are markedly worse for Black children compared to their White or Hispanic peers. This is likely due in part to the greater frequency and intensity of environmental Pb exposure for young Black children, while direct measurements of exposure to Pb were not examined in the current study that instead utilizes indirect measurements of Pb exposure risks. As mentioned earlier, previous research has demonstrated Black children are exposed to more Pb during early childhood, while an increasing and higher percent Black population is associated with greater amounts of environmental Pb exposure and more blood Pb among children. Higher airborne Pb emissions are correlated with increasing racial segregation of the Black population in the Detroit metropolitan area, which is a stronger predictor than poverty levels [48], while the authors also noted that unequal enforcement of environmental laws, emission standards, and remediation efforts by regulators in addition to facility siting preference by industry appear to play significant roles in these local racial disparities.

Black children experiencing higher rates of exposure to Pb during early childhood would help begin to account for the significant racial disparities we observed for blood Pb outcomes among risk factors related to housing conditions, socio-economic status, and younger age. In the current study, we observed that Black children from low-income households presented with far worse outcomes in continuous BLLs and EBLLs ≥5 µg/dL than their White or Hispanic peers in poverty (see Figure 2 and Figure 3 and Table 2). Unlike White or Hispanic children, blood Pb remained consistently higher for Black children after 1 year of age in which both mean BLLs and rates of an EBLL ≥5 µg/dL typically peak. Black children 3 years of age presented with more blood Pb than their 1-year-old White and Hispanic peers. We also found that Black children residing in older housing built during 1950 to 1977 have higher continuous BLLs and a similar risk of presenting with an EBLL ≥5 µg/dL compared to White or Hispanic children who reside in older housing built before 1950. These findings were surprising, as pre-1950 housing generally poses the greatest risk for increases in early childhood blood Pb which result from the higher likelihood for household Pb dust and deteriorating leaded paint hazards, soil contamination, and deteriorating leaded household service lines or plumbing fixtures still in use for delivering drinking water. In particular, greater Pb exposure from numerous environmental sources would help begin to explain why Black children passively exposed to tobacco smoke inside of their homes have a three times higher odds of presenting with an EBLL ≥5 µg/dL than their White or Hispanic peers who are also exposed to tobacco smoke—even after rigorous controlling for other risk factors, confounding variables, and even the average daily number of cigarettes smoked inside the house.

### 4.2. Safe Housing and Environmental Justice

Safe housing efforts that include the abatement, remediation, and prevention of environmental Pb hazards in older housing may therefore not be reaching young Black children in as equitable of a manner as their peers. Higher rates of substandard housing conditions afforded to Black families has previously been suggested to significantly contribute to early childhood blood Pb disparities [38,74]. Black children are more likely to reside in homes with leaded paint hazards and are exposed to more household Pb dust [67,68,69]. For households that rent, the Environmental Protection Agency (EPA) instructs tenants to notify their landlord about potential Pb hazards that include chipped or peeling paint. In some states, landlords are held liable for remediating leaded paint hazards under threat of fines [75]. Barriers that prevent tenants from raising concerns over leaded paint hazards include the fear of retaliation or eviction by their landlord. In particular, Black renter households continue to be more vulnerable to housing discrimination while enforcement of the Fair Housing Act remains very inconsistent between varying jurisdictions across the US [76]. Black households are twice as likely to rent compared to White households, while Black households face disproportionately higher eviction rates than White households in the same high-poverty income brackets [77]. Furthermore, geospatial analyses of demolition patterns in the predominantly Black cities of Baltimore and St. Louis reported significant associations with BLLs during early childhood [78,79], while higher demolition rates and vacant housing in predominantly Black neighborhoods are yet another racially disparate structural outcome of environmental injustice in the built environment for Black children.

Important programs for the prevention of early childhood Pb exposure in the US include the 1988 Childhood Lead Poisoning Prevention Program from the CDC to eliminate childhood exposure to Pb, in addition to the 1999 Healthy Homes Initiative (HHI) from the Department of Housing and Urban Development (HUD) that coordinates the identification and elimination of Pb hazards related to housing and childhood Pb exposure. Federally-owned or -subsidized housing must comply with stricter regulations of leaded paint hazards and their remediation under the 1999 Lead Safe Housing Rule by HUD, while children who reside in housing that receives public assistance from HUD at the national level or locally from the New York City Housing Authority (NYCHA) have significantly lower blood Pb [80,81]. Any alterations to buildings with leaded paint hazards are further regulated under the 1992 Renovation, Repair, and Painting Rule from the EPA. However, efforts in public health interventions and abatement present with racial disparities. Black children with identified EBLLs ≥10 µg/dL or higher consistently take far longer to fall below this threshold compared to their White or Hispanic peers even when controlling for their qualifying BLL and other variables [82]. In addition, follow-up BLLs continue to remain much higher for Black children following remediation efforts of household Pb hazards [83]. This may result in part from delayed timing in Pb abatement for Black children, lower follow-up rates for Black children, and lower rates of preventative pediatric screening services that are offered to Black children [82,83,84,85,86] although the exact causes of these racial disparities remain unknown and still require further research.

Other instances of environmental injustices may further contribute to our findings. In the State of New York, the two strongest predictors for early childhood EBLLs ≥10 µg/dL were zip codes with older housing or with a higher percentage of births to Black mothers [60]. Historical trends of blood Pb disparity also continue for Black women of childbearing age, those who are pregnant, and even their newborn children [87,88,89,90,91], while Black adults experience higher cumulative lifetime exposure to Pb [92]. After absorbed Pb is deposited in the bone mineral, it then later slowly leaches into the blood with increasing age or the loss of bone mineral density (BMD). Mobilization of calcium (Ca) from the maternal skeleton and transient BMD loss occur during pregnancy and breastfeeding, which results in the release of Pb deposited in the bone mineral and its subsequent transfer to the fetus or infants [93,94,95,96] who are both particularly vulnerable to the neurodevelopmental effects of Pb exposure. One recent study observed that the disparate Pb burden observed for Black children begins in utero and then persists into early childhood, which follows transmission from their Black mothers even after controlling for socio-economic or nutritional factors [91]. This represents a further intergenerational transmission of the historical and ongoing environmental injustice still suffered by Black mothers and passed on to their newborn Black infants who subsequently inherit this risk.

Therefore, Black racial disparities in early childhood blood Pb outcomes appear to result from a cumulative effect of numerous environmental injustices in the built environment that determine the markedly worse outcomes suffered by young Black children during their most critical stages of early childhood brain development. Given that even low-level blood Pb at levels of 2 to 3 µg/dL during early childhood are associated with a later loss of grade school IQ points [15,16,17] or the development of ADHD-like symptoms [18]–even slight increases amounting to a mean difference of 1 µg/dL for Black children compared to their peers can then have a substantially deleterious impact upon child developmental outcomes that result in expected social costs amounting to billions in $US dollars just from losses in lifetime earnings alone associated with IQ points lost to early childhood BLLs [17]. Furthermore, the greatest loss of grade school IQ points from early childhood BLLs occur with the first few point increases in blood Pb concentrations between 2 and 4 µg/dL, as this insidious and latent long-term effect associated with early childhood BLLs follows a log-linear trend. The elimination of Black racial disparity in early childhood blood Pb would require a far more multifaceted approach to combat the numerous varying and accumulating environmental injustices that are currently suffered by Black children with regards to Pb exposure, which then collectively result in the markedly worse blood Pb outcomes for Black children during the first few years of life when the most critical periods of neurodevelopment occur for highly vulnerable infants and preschool-aged children.

### 4.3. Targeted Screening and Public Health Implications

**Figure 4:**
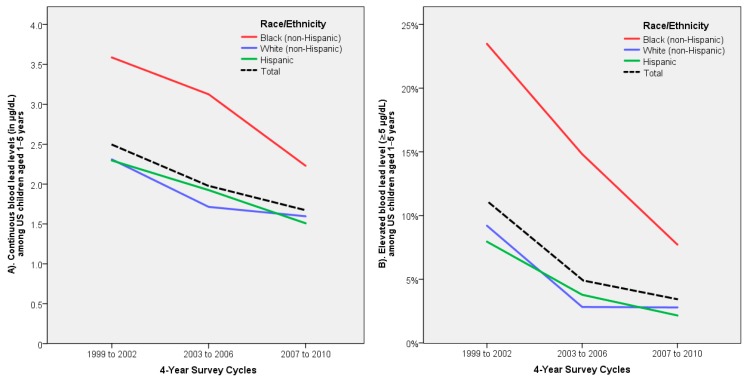
Weighted estimates of means for (**A**) continuous blood lead levels (BLLs) or (**B**) rates of elevated BLLs (EBLLs) ≥5 µg/dL among US children 1–5 years of age with US citizenship—the 1999 to 2010 NHANES.

Significant racial disparity in early childhood blood Pb for Black children has been documented in national surveys by the CDC since 1976 [24,25,26,27]. Despite efforts to target at-risk children in the US since the early 1990s, significant racial disparity has continued nationwide during the 1999 to 2010 survey periods (see Figure 4A,B), as reported in our study and others [41]. We observed that it had taken Black children approximately eight years longer to first reach the same declines in blood Pb during 2007 to 2010 that were already seen for White or Hispanic children during 1999 to 2002. In the State of Illinois, significant racial disparity for early childhood EBLLs ≥6 µg/dL observed among predominantly Black neighborhoods in the City of Chicago during 1995 were also observed later in 2010 but had become somewhat weaker [97]. Targeted screening of predominantly Black areas at high risk in Chicago, Illinois, during 2001 revealed that nearly two out of three children with an EBLL ≥10 µg/dL were not previously tested and therefore went unidentified until the study had examined them [98]. At the national level, efforts in targeted screening miss one in three children with an EBLL ≥10 µg/dL, while up to two out of three EBLL-positive children in the South are not identified [74]. These conditions disproportionately impact Black children, as they are exposed to greater amounts of environmental Pb and continue to have much higher BLLs than their White or Hispanic peers.

In spite of these well-known disparities in blood Pb for Black children, there are no indications for Black race in the more commonly used risk screening assessments for early childhood Pb such as the 1991 CDC questionnaire to identify EBLLs ≥10 µg/dL, recent recommendations for pediatricians from the AAP and ACPM, or many targeted screening programs conducted by differing states in the US. In the State of Tennessee, the strongest predictor of early childhood EBLLs ≥5 µg/dL is residing in census tracts with the highest quintile for percent Black populations that outperformed all other risk factors such as older housing, poverty, and younger age after adjustments in logistic regression [45]. Analysis of census tracts in the State of Maryland demonstrated that Black race was the second strongest predictor of an EBLL ≥5 µg/dL during early childhood after older housing built before 1940 [54], which is similar to our own findings regarding Black race and pre-1950 housing. In Maryland, the highest rates for an early childhood EBLL ≥5 µg/dL were largely clustered in the predominantly Black city of Baltimore. The authors had recommended that intervention efforts be targeted in areas with a higher percent Black population or pre-1940 housing in particular and then followed by areas with higher rates of low-income households or poverty to a lesser extent.

In the current study, we observed that blood Pb outcomes for risk factors of early childhood Pb exposure confer two markedly different conditions for Black children when compared to White or Hispanic children, which further raises important questions concerning the validity of their current application in risk screening efforts or when race is neglected as a key mediating factor. This may help explain why risk screening questionnaires from the CDC and others are found to perform only a little better than if left to pure chance alone [30,37]. Other risk screening questionnaires have more favorable performances, such as one by Kaplowitz et al. [33] in the State of Michigan that included Black race in their risk assessments as an individual- and ecological-level covariate—both of which were significant independent predictors of early childhood blood Pb. The studies from Michigan by Kaplowitz et al. [33], from Tennessee by Ford et al. [45], and from Maryland by Wheeler et al. [54] in addition to our own demonstrate the importance of both recognizing and incorporating Black race as a risk factor when screening for early childhood Pb exposure or blood Pb. However, even better performing criteria for Michigan by Kaplowitz et al. [33] would still miss an estimated 31% of all EBLL-positive children with a BLL ≥7 µg/dL who reside in the state. Although it is more costly than targeted screening, universal BLL testing during early childhood would close gaps in identification by detecting all cases that are EBLL-positive [99]. Universal testing of early childhood BLLs would also allow for a near-complete understanding of the remaining troubled areas of Pb contamination, continuing socio-economic or racial/ethnic disparity, and how to devise better performing targeting, preventive, intervention, and abatement or remediation efforts [100,101]. Furthermore, conservative estimates demonstrate that each $1 US dollar invested in controls just for leaded paint hazards alone generate a return of at least $17 USD, which then results in a net savings of $181 billion USD [102]. Lastly, universal testing of early childhood BLLs would greatly assist in identifying and eliminating the remaining hazards for Pb exposure in the US that are causing existing disparities for outcomes.

### 4.4. Study Limitations and Interpretation

There are certain limitations to our current study. The study period was limited to before 2011, as the year housing was built is only available for the 1999 to 2010 NHANES. Examining following years would require linking external data on housing to individual cases in the NHANES. Data on parental occupation that can result in take-home contamination [103], proximity to lead-emitting industry, leaded household plumbing, or Pb intake from drinking water are not examined by the NHANES. However, it can be reasonably assumed that most of the affected households with Pb-contaminated drinking water involve older homes built before 1978 [104], given that bans on leaded household plumbing began in 1986. Recent renovation or removal of old paint, leaded paint hazards in the house, and household Pb dust measurements were not included in our analyses as a result of reporting only until 2004. Other direct measurements of exposure to Pb are not available in the NHANES. Covariate data for the type of home such as a house or apartment were not included as a result of reporting in the NHANES only until 2006, which led us against including whether the home was owned or rented in our analyses. Geographical variables that involve census tracts are limited access data from the NHANES that are not publicly available. Therefore, we only examined individual-level characteristics and not ecological-level variables such as the percent Black population. Other geographical covariates in the NHANES such as differing US regions or urban vs. rural setting were also not examined, although authors have reported such significant variations in childhood blood Pb nationally and between different states [82,105,106,107,108,109].

Missing data in the current study predominantly involved respondents who were unsure what year their housing was built in. These cases were therefore not included to help prevent introducing a potential bias of undefined data, while exclusion of these cases disproportionately reduced blood Pb in the final studied sample—particularly for Black children. Our findings may therefore represent an underreporting of the true existing racial disparities. As shown in the supplementary data (see Table S2), Black children instead had a 4-fold higher odds of presenting with an EBLL ≥5 µg/dL when cases with unknown housing age were included in analysis, while racial disparity in BLLs increased only slightly by +0.03 up to 0.86 µg/dL. Significant racial disparities in blood Pb were observed in children with current or recent anemia (+1.26 µg/dL; AOR = 9.2; *p* = 0.018 respectively). However, anemia was not included as a selected risk factor for our main analyses of racial disparity resulting from its lower reporting (*n* = 149) and far larger 95% CI outcomes (+0.22 to +2.31 µg/dL; AOR = 1.5 to 57.9), as shown in the supplementary data (see Table S3). Nutrition during pregnancy and infancy has been reported to impact early childhood blood Pb [89,110,111,112]. Iron deficiency increases dietary absorption of Pb^2+^ and other toxic metals such as cadmium (Cd) through the upregulation of divalent metal transporter 1 (DMT1), while essential trace elements such as Ca, iron (Fe), and zinc (Zn) appear to compete with Pb^2+^ ions for gastrointestinal (GI) absorption. NHANES data of dietary intakes were not included in this study, as they further reduced the sample size by 8% and are only limited to 1 or 2 day intake totals. As shown in the supplementary data (see Table S4), racial disparity continued to significantly persist for Black children at similar rates when controlling for continuous daily dietary intakes of Ca, Fe, and Zn that were each significantly associated with continuous BLLs (*p* = 0.015 to 0.025) but not EBLLs ≥5 µg/dL (*p* = 0.202 to 0.248).

Other/Multiracial children were excluded from the study due to low reporting in the NHANES. Children without US citizenship were excluded from the study to examine racial disparity in more equal conditions between children who are all US nationals. This was also further enacted as a result of low reporting and since there were no Black children in the sample lacking US citizenship. Most importantly, we were unable to discern for Black race among Hispanic children, who account for approximately 2.5% of the national Hispanic population while another 6% are multiracial [113]. As shown in Table 1 and Figure 4A,B, both non-Hispanic White and Hispanic children were grouped together as they presented with similar blood Pb values that do not significantly differ from one another, which is consistent with reports from both the CDC and of this survey period [28,38,41]. Furthermore, this categorical classification was done to provide general values for predominantly African-American Black children (14%) compared to the vast majority of the remaining US pediatric population (79%) except for Other/Multiracial children (7%). It is also important to note that Black Hispanic individuals may report their own race/ethnicity only as Hispanic, even when they believe they are perceived as Black by others [114,115,116], which then raises potential methodological concerns.

Therefore, interpretations from our current study are limited in a few key respects. Firstly, our findings only apply to young children who are US nationals with citizenship. Migrant children who have recently arrived to the US are more likely to present with an EBLL ≥10 µg/dL, which is a result of leaving higher Pb exposure environments, while their BLLs decrease in relation to their increasing length of time that is spent residing inside the US [117,118]. Children in more vulnerable Hispanic subpopulations were not examined in our present study [119], which included a sample of Hispanic children who are predominantly Mexican-American (80.3%) or otherwise reported only as “Other Hispanic” (19.7%). We were unable to identify vulnerable racial/ethnic subpopulations of Hispanic children that include Black Hispanic children. As a result, our findings are primarily applicable to children who are either Hispanic or White US nationals compared to Black US nationals who are all predominantly African-American. Caution should be practiced when generalizing our findings for children residing in the US who are Black but not African-American, Hispanic children who are not Mexican-American or belong to a vulnerable Hispanic subpopulation, and children who are Asian, multiracial, belong to other racial/ethnic groups, lack US citizenship, or recently emigrated to the US.

Prospective studies would be able to overcome these limitations and allow for analysis of Black racial disparity in blood Pb among children who are Hispanic or belong to another ethnic group by categorizing both race and ethnicity separately. In previous surveys, the NHANES had categorized race and ethnicity as their own separate questions, but this was no longer included for continuous NHANES surveys beginning in 1999. This would allow for better examination of immigration and citizenship status among all racial/ethnic groups. Ecological-level variables including neighborhood characteristics and other social determinants of health in the built environment such as the percent Black population, socio-economic deprivation, and industrial emissions should also be examined to better understand the role of social and structural conditions that contribute to Black racial disparity in early childhood blood Pb. Furthermore, prospective studies of the 1999 to 2010 NHANES dataset could examine whether significant interactions occur between Black race and established factors related to housing or socio-economic conditions. This would assist in helping understand why racial disparity persists for Black children, whether similar racial disparities exist in the absence of risk factors, and whether these outcomes become worse as risk increases. In addition, issues with the high rate of missing data in the 1999 to 2010 NHANES dataset associated with unknown housing age can be addressed through the use of data imputation or the external linking of housing data in a prospective study. Lastly, either Monte Carlo simulation or the bootstrapping method would help address uncertainty in our results, while variances estimated from complex sampling would help produce better weighted estimates for the risk factor subgroups.

## 5. Conclusions

At the national level, young Black children who are predominantly African-American continue to present with the highest average BLLs and rates of an EBLL ≥5 µg/dL compared to their White or Hispanic peers. Black race is also the second strongest predictor for increased blood Pb during early childhood after pre-1950 housing risk. Furthermore, Black racial disparity continues to significantly persist within each examined risk factor for early childhood blood Pb related to housing conditions, socio-economic status, and younger age. These significant racial disparities still remained even after correcting for other risk factors and variables. Greater Pb exposure has repeatedly been documented among Black children, which likely contributes to these disparate outcomes. Therefore, Black racial disparity in early childhood blood Pb appears to reflect social and structural issues of inequity and inequality, particularly social determinants of health such as those related to environmental justice in safe housing and the built environment that are not reaching Black children nor predominantly Black areas in an equitable manner. Further study of the consequences from higher early childhood Pb exposure for Black children remains unexamined, including IQ point loss from early childhood BLLs or their associated social costs. More research is needed regarding why these racial disparities appear and why they have continued unabated into the present day. Both renewed efforts and new approaches will likely be required in the prevention, screening, and targeting of early childhood Pb exposure to finally close the continued racial disparity gap that has been observed for Black children for more than four decades. One of the first and most essential steps to combat Black racial disparity for early childhood blood Pb would be recognizing Black race as a risk factor and then implementing it accordingly in preventative, screening, intervention, remediation, and abatement efforts. As Black children still currently continue to present with the highest average BLLs, the overall remaining burden of blood Pb during early childhood continues to be largely accounted for by Black children. Therefore, much of the significant remaining reductions that are still possible for early childhood Pb exposure are disproportionately overrepresented by young Black children who are predominantly African-American. Black racial disparity in early childhood blood Pb has now stubbornly persisted throughout the US for over four decades in the face of a sustained grossly insufficient institutional response from the federal, state, and local levels.

## Figures and Tables

**Table 1:** Descriptive statistics and simple means for continuous blood lead levels (BLLs) or an elevated BLL (EBLL) ≥5 µg/dL sampled in multiple regression model analysis—the 1999 to 2010 NHANES.

Independent Variable	Reporting	Sample (%)	BLL (in µg/dL)		EBLL (≥5 µg/dL)	
			Mean ± SD	*p*-Value	Mean ± SE	*p*-Value
**Age in Years**	100%					
4–5 years		1028 (36.2%)	1.76 ± 1.40	–	3.2% ± 0.6%	–
3 years		463 (16.3%)	1.94 ± 1.60	0.121	5.2% ± 1.0%	0.092
2 years		685 (24.1%)	2.28 ± 2.23	<0.001 **	8.0% ± 1.0%	<0.001 **
1 year		665 (23.4%)	2.49 ± 2.97	<0.001 **	10.8% ± 1.2%	<0.001 **
**Anemia**	99.5%					
No		2692 (94.8%)	2.06 ± 2.05	–	6.1% ± 0.5%	–
Yes		149 (5.2%)	2.47 ± 3.02	0.101	12.8% ± 2.7%	0.001 **
**Binary Gender**	100%					
Female		1338 (47.1%)	2.05 ± 1.95	–	5.9% ± 0.6%	–
Male		1503 (52.9%)	2.11 ± 2.25	0.470	7.0% ± 0.7%	0.242
**Bodyweight (in kg)**	97.9%					
Continuous (scaled)		2841 (100%)	−0.11 per unit	<0.001 **	−0.09% per unit	<0.001 **
**Health Insurance Coverage**	99.3%					
Yes		2547 (89.7%)	2.05 ± 2.12	–	6.2% ± 0.5%	–
No		294 (10.3%)	2.37 ± 2.09	0.013 **	9.2% ± 1.7%	0.086
**Household Income Level**	93.6%					
Middle to upper class		1142 (40.2%)	1.64 ± 1.32	–	2.9% ± 0.5%	–
Low income		831 (29.3%)	2.19 ± 2.49	<0.001 **	7.2% ± 0.9%	<0.001 **
Poverty		868 (30.6%)	2.57 ± 2.44	<0.001 **	10.5% ± 1.0%	<0.001 **
**Indoor Household Smoking**	99.1%					
No		2392 (84.2%)	1.94 ± 2.02	–	5.2% ± 0.5%	–
Yes		449 (15.8%)	2.83 ± 2.46	<0.001 **	13.4% ± 1.6%	<0.001 **
**Low Birthweight**	99.7%					
No		2601 (91.6%)	2.06 ± 2.09	–	6.2% ± 0.5%	–
Yes		240 (8.4%)	2.31 ± 2.39	0.116	9.6% ± 1.9%	0.041 *
**Medicaid/CHIP Enrollment**	99.3%					
No		1934 (68.1%)	1.93 ± 2.16	–	5.4% ± 0.5%	–
Yes		907 (31.9%)	2.40 ± 1.98	<0.001 **	8.8% ± 0.9%	0.001 **
**Primary Guardian Education**	97.3%					
Some college or higher		1377 (48.5%)	1.90 ± 2.20	–	4.9% ± 0.6%	–
12th grade or GED		706 (24.9%)	2.10 ± 1.68	0.038 *	6.9% ± 1.0%	0.060
Less than 12th grade or GED		758 (26.7%)	2.40 ± 2.28	<0.001 **	8.8% ± 1.0%	<0.001 **
**Race/Ethnicity**	100%					
Hispanic		1025 (36.1%)	1.82 ± 1.71	–	4.4% ± 0.6%	–
NH White		1208 (42.5%)	1.94 ± 1.83	0.166	5.5% ± 0.7%	0.245
NH Black		608 (21.4%)	2.81 ± 2.95	<0.001 **	12.0% ± 1.3%	<0.001 **
**Survey Years**	100%					
1999 to 2002		832 (29.3%)	2.56 ± 2.82	–	10.9% ± 1.1%	–
2003 to 2006		1035 (36.4%)	2.06 ± 1.80	<0.001 **	5.6% ± 0.7%	<0.001 **
2007 to 2010		974 (34.3%)	1.70 ± 1.59	<0.001 **	3.6% ± 0.6%	<0.001 **
**Water Treatment Devices Used**	98.7%					
No		2108 (74.2%)	2.23 ± 2.33	–	7.5% ± 0.6%	–
Yes		733 (25.8%)	1.67 ± 1.25	<0.001 **	3.5% ± 0.7%	**<** 0.001 **
**WIC Enrollment**	99.5%					
No		1644 (57.9%)	1.90 ± 2.11	–	4.8% ± 0.5%	–
Yes		1197 (42.1%)	2.33 ± 2.10	**<** 0.001 **	8.8% ± 0.8%	**<** 0.001 **
**Year Housing Built**	64.4%					
1978 to present		1282 (45.1%)	1.66 ± 1.26	–	2.9% ± 0.5%	–
1950 to 1977		945 (33.3%)	2.04 ± 1.76	<0.001 **	5.3% ± 0.7%	0.004 **
Before 1950		614 (21.6%)	3.03 ± 3.38	<0.001 **	15.8% ± 1.5%	<0.001 **
**TOTAL**	59.6%	2841	2.08 ± 2.12	–	6.5% ± 0.5%	–

**Statistical significance:** * *p*-value equal to 5% or less; ** *p*-value equal to 1% or less.

**Table 2:** Continuous blood lead levels (BLLs) or an elevated BLL (EBLL) ≥5 µg/dL after correcting for risk factors and other covariates in full multiple regression model analysis—the 1999 to 2010 NHANES.

Independent Variable	BLL (in µg/dL)			EBLL (≥5 µg/dL)		
	B Coeff. (β)	95% CI	*p*-Value	Odds Ratio	95% CI	*p*-Value
**Age in Years**						
4–5 years	Reference	–	–	Reference	–	–
3 years	+0.19 per unit (+0.105)	+0.10 to +0.28	<0.001 **	1.7	0.92 to 3.2	0.089
2 years				2.7	1.5 to 4.9	0.001 **
1 year				3.4	1.7 to 6.9	0.001 **
**Anemia**						
No	Reference	–	–	Reference	–	–
Yes	+0.29 (+0.031)	−0.03 to +0.61	0.074	2.2	1.3 to 4.0	0.005 **
**Binary Gender**						
Female	Reference	–	–	Reference	–	–
Male	+0.11 (+0.025)	−0.04 to +0.25	0.151	1.3	0.90 to 1.8	0.178
**Bodyweight (in kg)**						
Continuous (scaled)	−0.02 per unit (−0.050)	−0.05 to 0.00	0.051 *	0.98	0.92 to 1.1	0.554
**Health Insurance Coverage**						
Yes	Reference	–	–	Reference	–	–
No	+0.26 (+0.038)	+0.01 to +0.52	0.039 *	1.3	0.77 to 2.2	0.332
**Household Income Level**						
Middle to upper class	Reference	–	–	Reference	–	–
Low income	+0.28 per unit (+0.109)	+0.17 to +0.39	<0.001 **	2.2	1.4 to 3.7	0.002 **
Poverty				2.5	1.5 to 4.2	0.001 **
**Indoor Household Smoking**						
No	Reference	–	–	Reference	–	–
Yes	+0.42 (+0.072)	+0.21 to +0.62	<0.001 **	1.8	1.2 to 2.7	0.002 **
**Low Birthweight**						
No	Reference	–	–	Reference	–	–
Yes	+0.05 (+0.007)	−0.21 to +0.31	0.681	1.3	0.79 to 2.3	0.285
**Medicaid/CHIP Enrollment**						
No	Reference	–	–	Reference	–	–
Yes	+0.12 (+0.027)	−0.07 to +0.31	0.204	1.0	0.70 to 1.5	0.859
**Primary Guardian Education**						
Some college or higher	Reference	–	–	Reference	–	–
12th grade or GED	+0.11 per unit (+0.044)	+0.01 to +0.21	0.026 *	1.0	0.65 to 1.6	0.986
Less than 12th grade or GED				1.3	0.86 to 2.0	0.210
**Race/Ethnicity**						
Hispanic	Reference	–	–	Reference	–	–
NH White	+0.46 per unit (+0.161)	+0.36 to +0.56	<0.001 **	1.5	0.97 to 2.4	0.070
NH Black				3.5	2.2 to 5.5	<0.001 **
**Survey Years**						
1999 to 2002	Reference	–	–	Reference	–	–
2003 to 2006	−0.40 per unit (−0.151)	−0.49 to −0.31	<0.001 **	0.42	0.29 to 0.62	<0.001 **
2007 to 2010				0.31	0.20 to 0.48	<0.001 **
**Water Treatment Devices Used**						
No	Reference	–	–	Reference	–	–
Yes	−0.21 (−0.043)	−0.38 to −0.04	0.015 *	0.83	0.52 to 1.3	0.420
**WIC Enrollment**						
No	Reference	–	–	Reference	–	–
Yes	−0.01 (−0.003)	−0.19 to +0.16	0.889	1.0	0.70 to 1.5	0.908
**Year Housing Built**						
1978 to present	Reference	–	–	Reference	–	–
1950 to 1977	+0.61 per unit (+0.226)	+0.52 to +0.70	<0.001 **	1.9	1.2 to 2.9	0.007 **
Before 1950				7.1	4.6 to 10.8	<0.001 **

**Statistical significance:** * *p*-value equal to 5% or less; ** *p*-value equal to 1% or less.

**Table 3:** Racial disparities in blood lead (Pb) level outcomes for risk factor groups of early childhood Pb exposure before and after correcting for covariates in multiple regression model analysis—the 1999 to 2010 NHANES.

Model	Independent Variable	Sample (%)	BLL (in µg/dL)				EBLL (≥5 µg/dL)			
*Risk Factor*			Mean ± SD	B Coeff. (β)	95% CI	*p*-Value	Mean ± SE	Odds Ratio	95% CI	*p*-Value
**Full Model** Total Sample	NH White or Hispanic	2233 (78.6%)	1.89 ± 1.78	Reference	–	–	5.0% ± 0.5%	Reference	–	–
	NH Black	608 (21.4%)	2.81 ± 2.95	+0.92 (+0.179)	+0.74 to +1.11	<0.001 **	12.0% ± 1.3%	2.6	1.9 to 3.6	<0.001 **
	*Adjusted* ^a^			+0.83 (+0.160)	+0.65 to +1.00	<0.001 **		2.8	1.9 to 3.9	<0.001 **
**Model 1—Age** 1 Year of Age	NH White or Hispanic	526 (79.1%)	2.22 ± 2.34	Reference	–	–	8.9% ± 1.2%	Reference	–	–
	NH Black	139 (20.9%)	3.51 ± 4.53	+1.29 (+0.177)	+0.74 to +1.84	<0.001 **	18.0% ± 3.3%	2.2	1.3 to 3.8	0.003 **
	*Adjusted* ^a,b^			+1.00 (+0.136)	+0.47 to +1.53	<0.001 **		2.0	1.1 to 3.7	0.035 *
**Model 2—Age** 2 Years of Age	NH White or Hispanic	554 (80.8%)	2.00 ± 1.84	Reference	–	–	5.2% ± 0.9%	Reference	–	–
	NH Black	131 (19.2%)	3.45 ± 3.19	+1.45 (+0.256)	+1.04 to +1.86	<0.001 **	19.8% ± 3.5%	4.5	2.5 to 7.9	0.001 **
	*Adjusted* ^a,b^			+1.41 (+0.249)	+1.01 to +1.81	<0.001 **		5.3	2.6 to 10.7	<0.001 **
**Model 3—Age** 3 Years of Age	NH White or Hispanic	355 (76.7%)	1.75 ± 1.47	Reference	–	–	3.7% ± 1.0%	Reference	–	–
	NH Black	108 (23.3%)	2.55 ± 1.87	+0.80 (+0.211)	+0.46 to +1.14	<0.001 **	10.2% ± 2.9%	3.0	1.3 to 6.9	0.007 **
	*Adjusted* ^a,b^			+0.73 (+0.192)	+0.40 to +1.06	<0.001 **		4.7	1.7 to 13.1	0.003 **
**Model 4—Education** Primary Guardian <12th Grade/GED	NH White or Hispanic	624 (82.3%)	2.17 ± 2.12	Reference	–	–	6.3% ± 1.0%	Reference	–	–
	NH Black	134 (17.7%)	3.49 ± 2.67	+1.32 (+0.221)	+0.90 to +1.74	<0.001 **	20.9% ± 3.5%	4.0	2.3 to 6.7	<0.001 **
	*Adjusted* ^a,c^			+0.99 (+0.166)	+0.58 to +1.40	<0.001 **		3.5	1.8 to 6.7	<0.001 **
**Model 5—Housing** Housing Built 1950 to 1977	NH White or Hispanic	727 (76.9%)	1.78 ± 1.36	Reference	–	–	3.0% ± 0.6%	Reference	–	–
	NH Black	218 (23.1%)	2.90 ± 2.52	+1.12 (+0.268)	+0.86 to +1.38	<0.001 **	12.8% ± 2.3%	4.7	2.6 to 8.4	<0.001 **
	*Adjusted* ^a,d^			+1.02 (+0.244)	+0.76 to +1.27	<0.001 **		5.6	2.8 to 11.2	<0.001 **
**Model 6—Housing** Housing Built <1950	NH White or Hispanic	506 (82.4%)	2.73 ± 2.71	Reference	–	–	13.4% ± 1.5%	Reference	–	–
	NH Black	108 (17.6%)	4.43 ± 5.32	+1.70 (+0.192)	+1.01 to +2.40	<0.001 **	26.9% ± 4.3%	2.4	1.4 to 3.9	0.001 **
	*Adjusted* ^a,d^			+1.22 (+0.138)	+0.54 to +1.90	<0.001 **		1.8	1.0 to 3.2	0.053 *
**Model 7—Housing** Indoor Household Smoking	NH White or Hispanic	311 (71.3%)	2.45 ± 1.95	Reference	–	–	9.6% ± 1.7%	Reference	–	–
	NH Black	122 (28.7%)	3.93 ± 3.30	+1.48 (+0.268)	+0.98 to +1.99	<0.001 **	24.6% ± 3.9%	3.1	1.8 to 5.4	<0.001 **
	*Adjusted* ^a,e^			+1.30 (+0.234)	+0.79 to +1.80	<0.001 **		3.3	1.6 to 6.4	0.001 **
**Model 8—Income** Low Income	NH White or Hispanic	674 (81.1%)	2.01 ± 1.93	Reference	–	–	6.2% ± 0.9%	Reference	–	–
	NH Black	157 (18.9%)	2.96 ± 4.03	+0.94 (+0.148)	+0.51 to +1.37	<0.001 **	11.5% ± 2.6%	2.0	1.1 to 3.5	0.025 *
	*Adjusted* ^a,f^			+0.90 (+0.142)	+0.48 to +1.32	<0.001 **		2.1	1.1 to 4.1	0.034 *
**Model 9—Income** Poverty	NH White or Hispanic	647 (74.5%)	2.24 ± 2.07	Reference	–	–	7.1% ± 1.0%	Reference	–	–
	NH Black	221 (25.5%)	3.53 ± 3.10	+1.29 (+0.231)	+0.93 to +1.65	<0.001 **	20.4% ± 2.7%	3.3	2.1 to 5.2	<0.001 **
	*Adjusted* ^a,f^			+1.22 (+0.220)	+0.89 to +1.58	<0.001 **		4.1	2.3 to 7.0	<0.001**

**Covariates:**^a^ examined risk factors; survey years; binary gender; bodyweight; low birthweight; anemia; health insurance coverage; Medicaid/CHIP enrollment; federal Women, Infants, and Children (WIC) supplemental food program enrollment; use of water treatment devices; ^b^ age in months; ^c^ educational attainment less than 9th grade education; ^d^ housing built before 1960 or 1940; ^e^ number of cigarettes smoked inside the home per day (1 to 40 or more); ^f^ poverty-to-income ratios. **Statistical significance:** * *p*-value equal to 5% or less; ** *p*-value equal to 1% or less.

## Supplementary Materials

The following are available online at https://www.mdpi.com/1660-4601/17/5/1552/s1. Table S1: Racial/ethnic differences in continuous BLLs or an EBLL ≥5 µg/dL before and after the exclusion of cases with missing data; Table S2: Continuous BLLs or an elevated EBLL ≥5 µg/dL after inclusion of unknown housing age in multiple regression analysis; Table S3: Continuous BLLs or an EBLL ≥5 µg/dL among children with current or recent anemia in multiple regression analysis; Table S4: Continuous BLLs or an EBLL ≥5 µg/dL after inclusion of dietary Ca, Fe, and Zn intake in multiple regression analysis.
