# Supplementary material for: Disparity in Risk Factor Severity for Early Childhood Blood Lead among Predominantly African-American Black Children: The 1999 to 2010 US NHANES
Source: Int J Environ Res Public Health. 2020 Feb 28;17(5):1552. doi: 10.3390/ijerph17051552 (PMC7084658; doi:10.3390/ijerph17051552)
Supplement: Supplementary file 1 [file ijerph-17-01552-s001.pdf]

Table S1. Racial/ethnic differences in continuous BLLs or an EBLL ≥5 μg/dL before and after the exclusion of cases with missing data.

Race/Ethnicity	Sampled Data	Sample (%)	BLL (in μg/dL)	EBLL (≥5 μg/dL)
			Mean **±** ***SD***	***Mean*** **±** ***SE***
Hispanic	Total	1954 (41.0%)	1.96 ± 1.81	5.0% ± 0.5%
	Modeled sample	1025 (36.1%)	1.82 ± 1.71	4.4% ± 0.6%
	Excluded cases	929 (48.2%)	2.12 ± 1.91	5.6% ± 0.8%
NH White	Total	1519 (31.8%)	2.01 ± 1.83	6.1% ± 0.6%
	Modeled sample	1208 (42.5%)	1.94 ± 1.83	5.5% ± 0.7%
	Excluded cases	311 (16.1%)	2.26 ± 1.80	8.7% ± 1.6%
NH Black	Total	1297 (27.2%)	3.18 ± 3.55	17.2% ± 1.0%
	Modeled sample	608 (21.4%)	2.81 ± 2.95	12.0% ± 1.3%
	Excluded cases	689 (35.7%)	3.51 ± 3.99	21.8% ± 1.6%
Total	Total	4770 (100%)	2.31 ± 2.48	8.7% ± 0.4%
	Modeled sample	2841 (59.6%)	2.08 ± 2.12	6.5% ± 0.5%
	Excluded cases	1929 (40.4%)	2.64 ± 2.89	11.9% ± 0.7%

Table S2. Continuous BLLs or an elevated EBLL ≥5 μg/dL after inclusion of unknown housing age in multiple regression analysis.

Independent Variable	BLL (in μg/dL)			EBLL (≥5 μg/dL)		
	B Coeff. (β)	95% CI	*p*-Value	Odds Ratio	95% CI	*p*-Value
**Age in Years**						
4–5 years	Reference	–	–	Reference	–	–
3 years	+0.16 per unit (+0.083)	+0.08 to +0.23	**<0.001****	1.3	0.85 to 2.0	0.218
2 years				1.8	1.2 to 2.8	**0.007****
1 year				1.9	1.2 to 3.2	**0.012****
**Anemia**						
No	Reference	–	–	Reference	–	–
Yes	+0.08 (+0.008)	–0.18 to +0.34	0.557	1.2	0.80 to 1.9	0.330
**Binary Gender**						
Female	Reference	–	–	Reference	–	–
Male	+0.11 (+0.025)	–0.01 to +0.24	0.082	1.3	0.98 to 1.6	0.070
**Bodyweight (in kg)**						
(scaled)	–0.03 (–0.061)	–0.05 to –0.01	**0.004****	0.95	0.91 to 0.998	**0.042***
**Health Insurance Coverage**						
Yes	Reference	–	–	Reference	–	–
No	+0.18 (+0.026)	–0.03 to +0.39	0.086	1.3	0.85 to 1.9	0.235
**Household Income Level**						
Middle to upper class	Reference	–	–	Reference	–	–
Low income	+0.23 per unit (+0.088)	+0.14 to +0.33	**<0.001****	1.8	1.2 to 2.7	**0.005****
Poverty				2.2	1.5 to 3.4	**<0.001****
**Indoor Household Smoking**						
No	Reference	–	–	Reference	–	–
Yes	+0.62 (+0.106)	+0.45 to +0.79	**<0.001****	2.0	1.5 to 2.6	**<0.001****
**Low Birthweight**						
No	Reference	–	–	Reference	–	–
Yes	+0.14 (+0.018)	–0.07 to +0.35	0.202	1.5	1.1 to 2.1	**0.024***
**Medicaid/CHIP Enrollment**						
No	Reference	–	–	Reference	–	–
Yes	+0.04 (+0.009)	–0.11 to +0.20	0.599	1.1	0.86 to 1.5	0.369
**Primary Guardian Education**						
Some college or higher	Reference	–	–	Reference	–	–
12th grade or GED	+0.08 per unit (+0.031)	–0.002 to +0.16	0.056	1.1	0.76 to 1.5	0.732
Less than 12th grade or GED				1.3	0.91 to 1.7	0.164
**Race/Ethnicity**						
NH White or Hispanic	Reference	–	–	Reference	–	–
NH Black	+0.89 (+0.179)	+0.75 to +1.04	**<0.001****	3.6	2.8 to 4.6	**<0.001****
**Survey Years**						
1999 to 2002	Reference	–	–	Reference	–	–
2003 to 2006	–0.41 per unit (–0.147)	–0.49 to –0.33	**<0.001****	0.45	0.34 to 0.59	**<0.001****
2007 to 2010				0.32	0.23 to 0.44	**<0.001****
**Water Treatment Devices Used**						
No	Reference	–	–	Reference	–	–
Yes	–0.12 (–0.023)	–0.28 to +0.04	0.130	0.89	0.61 to 1.3	0.558
**WIC Enrollment**						
No	Reference	–	–	Reference	–	–
Yes	–0.08 (–0.018)	–0.22 to +0.07	0.284	0.92	0.70 to 1.2	0.540
**Year Housing Built**						
1978 to present	Reference	–	–	Reference	–	–
1950 to 1977	+0.37 per unit (+0.175)	+0.31 to +0.43	**<0.001****	1.8	1.2 to 2.9	**0.009****
Unknown				2.6	1.8 to 3.9	**<0.001****
Before 1950				7.4	4.8 to 11.2	**<0.001****

Statistical significance: * P-value equal to 5% or less; ** P-value equal to ≤1% or less.

Table S3. Continuous BLLs or an EBLL ≥5 μg/dL among children with current or recent anemia in multiple regression analysis.

Independent Variable	BLL (in μg/dL)			EBLL (≥5 μg/dL)		
	B Coeff. (β)	95% CI	*p*-Value	Odds Ratio	95% CI	*p*-Value
**Age in Years**						
4–5 years	Reference	–	–	Reference	–	–
3 years	+0.76 per unit (+0.295)	+0.14 to +1.38	**0.016***	40	0.48 to 3400	0.102
2 years				79	0.82 to 7600	0.061
1 year				1200	3.1 to 450,000	**0.020***
**Binary Gender**						
Female	Reference	–	–	Reference	–	–
Male	–0.42 (–0.069)	–1.36 to +0.52	0.378	0.63	0.12 to 3.3	0.578
**Bodyweight (in kg)**						
(scaled)	+0.09 (+0.136)	–0.07 to +0.25	0.259	1.2	0.76 to 1.8	0.484
**Health Insurance Coverage**						
Yes	Reference	–	–	Reference	–	–
No	+1.18 (+0.128)	+0.34 to +2.70	0.126	22	1.9 to 270	**0.014****
**Household Income Level**						
Middle to upper class	Reference	–	–	Reference	–	–
Low income	+0.09 per unit (+0.026)	–0.60 to +0.78	0.799	1.3	0.12 to 14	0.831
Poverty				0.93	0.10 to 8.4	0.948
**Indoor Household Smoking**						
No	Reference	–	–	Reference	–	–
Yes	–0.37 (–0.043)	–1.70 to 0.96	0.585	0.27	0.03 to 2.6	0.258
**Low Birthweight**						
No	Reference	–	–	Reference	–	–
Yes	+0.99 (+0.078)	–0.96 to +2.94	0.318	33	1.3 to 830	**0.033***
**Medicaid/CHIP Enrollment**						
No	Reference	–	–	Reference	–	–
Yes	+0.44 (+0.071)	–0.69 to 1.56	0.445	15	1.7 to 130	**0.015***
**Primary Guardian Education**						
Some college or higher	Reference	–	–	Reference	–	–
12th grade or GED	+0.40 per unit (+0.114)	–0.19 to 1.00	0.184	0.33	0.04 to 2.8	0.306
Less than 12th grade or GED				0.71	0.11 to 4.7	0.716
**Race/Ethnicity**						
NH White or Hispanic	Reference	–	–	Reference	–	–
NH Black	+1.26 (+0.191)	+0.22 to +2.31	**0.018***	9.2	1.5 to 58	**0.018***
**Survey Years**						
1999 to 2002	Reference	–	–	Reference	–	–
2003 to 2006	–0.76 per unit (–0.211)	–1.31 to –0.21	**0.007****	0.61	0.12 to 3.1	0.547
2007 to 2010				0.12	0.02 to 0.92	**0.041***
**Water Treatment Devices Used**						
No	Reference	–	–	Reference	–	–
Yes	–0.25 (–0.035)	–1.37 to –0.87	0.661	0.31	0.04 to 2.5	0.266
**WIC Enrollment**						
No	Reference	–	–	Reference	–	–
Yes	+0.25 (+0.042)	–0.77 to +1.27	0.624	0.28	0.05 to 1.5	0.275
**Year Housing Built**						
1978 to present	Reference	–	–	Reference	–	–
1950 to 1977	+1.29 per unit (+0.324)	+0.67 to 1.91	**<0.001****	5.1	0.81 to 32	0.083
Before 1950				27	3.1 to 230	**0.003****

Statistical significance: * P-value equal to 5% or less; ** P-value equal to ≤1% or less.

Table S4. Continuous BLLs or an EBLL ≥5 μg/dL after inclusion of dietary Ca, Fe, and Zn intake in multiple regression analysis.

Independent Variable	BLL (in μg/dL)			EBLL (≥5 μg/dL)		
	B Coeff. (β)	95% CI	*p*-Value	Odds Ratio	95% CI	*p*-Value
**Age in Years**						
4–5 years	Reference	–	–	Reference	–	–
3 years	+0.17 per unit (+0.103)	+0.08 to +0.25	**<0.001****	1.9	0.99 to 3.6	**0.054***
2 years				2.7	1.4 to 5.2	**0.002****
1 year				3.8	1.8 to 8.0	**<0.001****
**Anemia**						
No	Reference	–	–	Reference	–	–
Yes	+0.24 (+0.028)	–0.06 to +0.54	0.116	2.0	1.1 to 3.8	**0.028***
**Binary Gender**						
Female	Reference	–	–	Reference	–	–
Male	+0.11 (+0.029)	–0.02 to +0.25	0.100	1.4	0.97 to 1.1	0.076
**Bodyweight (in kg)**						
(scaled)	–0.02 (–0.051)	–0.043 to –0.0001	**0.049***	0.998	0.94 to 1.1	0.952
**Dietary Ca (in mg)**						
(scaled)	–0.0002 (–0.055)	–0.0004 to –0.00004	**0.015***	0.9997	0.999 to 1.0002	0.238
**Dietary Fe (in mg)**						
(scaled)	–0.02 (–0.053)	–0.03 to –0.002	**0.025***	0.97	0.94 to 1.01	0.202
**Dietary Zn (in mg)**						
(scaled)	+0.03 (+0.064)	+0.01 to +0.06	**0.020***	1.04	0.97 to 1.1	0.248
**Health Insurance Coverage**						
Yes	Reference	–	–	Reference	–	–
No	+0.19 (+0.030)	–0.04 to +0.43	0.109	1.2	0.67 to 2.1	0.548
**Household Income Level**						
Middle to upper class	Reference	–	–	Reference	–	–
Low income	+0.23 per unit (+0.097)	+0.12 to +0.33	**<0.001****	2.0	1.1 to 3.3	**0.014****
Poverty				2.3	1.3 to 4.0	**0.003****
**Indoor Household Smoking**						
No	Reference	–	–	Reference	–	–
Yes	+0.55 (+0.103)	+0.35 to +0.74	**<0.001****	2.0	1.4 to 3.1	**0.001****
**Low Birthweight**						
No	Reference	–	–	Reference	–	–
Yes	+0.07 (+0.009)	–0.18 to +0.31	0.597	1.4	0.83 to 2.5	0.192
**Medicaid/CHIP Enrollment**						
No	Reference	–	–	Reference	–	–
Yes	+0.17 (+0.041)	–0.004 to +0.35	0.056	1.1	0.72 to 1.7	0.673
**Primary Guardian Education**						
Some college or higher	Reference	–	–	Reference	–	–
12th grade or GED	+0.08 per unit (+0.036)	–0.01 to +0.17	0.074	0.87	0.55 to 1.4	0.555
Less than 12th grade or GED				1.3	0.85 to 2.1	0.216
**Race/Ethnicity**						
NH White or Hispanic	Reference	–	–	Reference	–	–
NH Black	+0.73 (+0.154)	+0.56 to +0.91	**<0.001****	3.0	2.0 to 4.4	**<0.001****
**Survey Years**						
1999 to 2002	Reference	–	–	Reference	–	–
2003 to 2006	–0.36 per unit (–0.151)	–0.45 to –0.28	**<0.001****	0.45	0.30 to 0.67	**<0.001****
2007 to 2010				0.30	0.19 to 0.49	**<0.001****
**Water Treatment Devices Used**						
No	Reference	–	–	Reference	–	–
Yes	–0.15 (–0.035)	–0.31 to +0.01	0.062	0.76	0.46 to 1.3	0.283
**WIC Enrollment**						
No	Reference	–	–	Reference	–	–

Yes	–0.05 (–0.012)	–0.21 to +0.12	0.567	1.01	0.68 to 1.5	0.959
**Year Housing Built**						
1978 to present	Reference	–	–	Reference	–	–
1950 to 1977	+0.60 per unit	+0.52 to +0.69	**<0.001****	1.9	1.2 to 3.1	**0.007****
Before 1950	(+0.243)			8.1	5.1 to 12.7	**<0.001****

Statistical significance: * P-value equal to 5% or less; ** P-value equal to ≤1% or less.
